# History, Present, and Progress of Frontotemporal Dementia in China: A Systematic Review

**DOI:** 10.1155/2012/587215

**Published:** 2012-03-25

**Authors:** Ru-Jing Ren, Yue Huang, Gang Xu, Chun-Bo Li, Qi Cheng, Sheng-Di Chen, Gang Wang

**Affiliations:** ^1^Department of Neurology and Institute of Neurology, Ruijin Hospital, Shanghai Jiao Tong University School of Medicine, Shanghai 200025, China; ^2^Neuroscience Research Australia and the University of New South Wales, Randwick, NSW 2052, Australia; ^3^Department of Epidemiology, Public Health School, Shanghai Jiao Tong University School of Medicine, Shanghai 200025, China; ^4^Shanghai Mental Health Center, Shanghai Jiao Tong University School of Medicine, Shanghai 200025, China

## Abstract

We aim to provide an overview of clinical and demographical features and neuropathological research on frontotemporal dementia (FTD) from China over the past decade. We reviewed the demographic features, clinical presentations, and neuropathology of the FTD-spectrum disorders from the 49 cases in China published since 1998. On the basis of these findings, we retrospect the history and speculate on future progress in terms of FTD in China. We found that most published papers comprise case reports with a few retrospective studies with small sample sizes. Behavior variant FTD (bvFTD) was the most common diagnostic subtype, of which 35% were associated with amyotrophic lateral sclerosis or Parkinsonian syndrome. More than 47% patients with FTD had age onset before 65. There were no differences in age of onset and sex distribution between diagnostic subtypes. The spectrum of neuropathological diagnosis of bvFTD was frontotemporal lobe degeneration (FTLD) with tau protein or ubiquitin-immunopositive inclusions, and FTLD without intracellular inclusions. Median survival in bvFTD was 14 years. This paper provides an overview of the current status and pointers for future research directions of FTD in China.

## 1. Introduction

Frontotemporal dementia (FTD) is a clinically and pathologically heterogeneous syndrome, characterized by progressive decline in behavior or language functions associated with frontal and temporal lobe degeneration (FTLD) [1–4]. Although FTD was first described in the early 1900s as an early-onset disorder presenting before the age of 65, it has only recently been appreciated as a leading cause of dementia, and to be more common than previously recognized in advanced age [5]. Extensive worldwide studies on FTD have been conducted, including epidemiological investigations, cohort, and retrospective studies [6–8]. An interesting study, in terms of the distribution of FTD across four ethnic groups, claimed that it was as common in Asians as Caucasians [9]. Unfortunately, the epidemiological and demographic characteristics of FTD in China have not been investigated to date, where millions of patients suffer from Alzheimer's disease (AD) [10, 11]. Given that FTD is the second most common early-onset dementia after AD in the west [1–4], the epidemiology of FTD of China is of considerable interest.

Since the introduction of standardized clinical diagnostic criteria for FTD [12] into China, there has been an increasing interest in each of the three clinical variants: bvFTD [13–28], semantic dementia (SD) [29–31], and progressive noninfluent aphasia (PFNA) [32–37]. However, the level of research has been limited to case reports and occasional retrospective studies with small sample size. Diagnostic accuracy remains a problem, and there is a lack of collaboration between study centers or sites [27]. As a result, the profile of FTD in China remains obscure compared to the western world.

We reviewed the demographic features, clinical presentations, and neuropathology of the FTD-spectrum disorders from the 49 cases in China published since 1998. On the basis of these findings, we speculate on future progress in China and highlight the importance of international collaboration.

## 2. Methods

### 2.1. Search Strategy

We first searched the Cochrane Library (issue 1, 2011) for all systematic reviews that included cases of Chinese FTD. Subsequently, we searched MEDLINE (1948 to present), EMBASE (1980 to present), PsychInFO (1987 to present), and Chinese main medical databases: Chongqing VIP Database (VIP), Wan Fang Database, Chinese hospital knowledge database (CHKD), and Chinese biomedical database (CBM-disc, 1978 to present) were searched to locate all case reports or case series published in the last 12 years (1998–2010) associated with FTD. The key words used in the search included “frontotemporal lobar degeneration,” “frontotemporal dementia,” “Pick disease,” “progressive non-influent aphasia,” “semantic dementia,” “primary progressive aphasia,” and intervention-specific terms such as “China” and “Chinese.” Every paper relating FTD was reviewed, and the reference lists were also examined to identify possible missed cases. No language restriction was applied.

### 2.2. Selection Criteria

Cases were included in this analysis if patients satisfied the clinical diagnosis criteria of any of the three variants of FTD [12]. For bvFTD, these include (1) insidious onset and gradual progression; (2) early decline in social interpersonal conduct; (3) early impairment in regulation of personal conduct; (4) early emotional blunting; (5) early loss of insight. Patients met the criteria for semantic dementia if they exhibited (1) insidious onset and gradual progression; (2) a language disorder characterized by empty fluent speech, loss of word meaning, or semantic paraphasias; (3) a perceptual disorder characterized by impaired recognition of familiar faces or object identity; (4) preserved perceptual matching and drawing reproduction; (5) preserved single-word repetition; (6) preserved ability to read aloud and write to dictation orthographically regular words. Finally, patients meet the criteria for PNFA if they had (1) insidious and gradual progression and (2) nonfluent spontaneous speech with at least one of agrammatism, phonemic paraphasias, or anomia. Patients were diagnosed as probable or possible amyotrophic lateral sclerosis (ALS) according to the El Escorial criteria [38]. Our exclusion criteria were if (1) the article had very limited information for FTD-related studies [39, 40]; (2) the patient was not Han Chinese ethnicity [41]. Duplicated cases were only counted once, if it appeared in several publications [42–45]. Overall, 49 cases from 25 publications were included in this report.

### 2.3. Data Collection and Analysis

For each individual case of FTD, the variables collected included age, gender, ethnicity, family history of dementia, concomitant disorders, Mini-Mental State Examination (MMSE) scores, and neuropathological diagnosis. Descriptive analysis was performed to examine the distribution of demographic characteristics. Categorical variables were compared using chi-square test, while ANOVA was used for continuous variables. Kaplan-Meier survival curve was used to assess the cumulative survival rate of FTD.

## 3. Results

### 3.1. Diagnostic Subgroups

From January 1, 1998 to December 31, 2010, 49 patients of Han Chinese descent were diagnosed as having FTD across Northern and Eastern China. The cases first presented to memory clinics or department of neurology (43/49); psychiatric clinics (3/49); GPs (general practitioners); others (3/49).

Overall, bvFTD was the most common diagnostic subgroup and accounted for 71.4% of all diagnoses (*n* = 35). PNFA was the second most common (*n* = 8), followed by SD (*n* = 6). Of the bvFTD patients, 17.1% (*n* = 6) and 20.0% (*n* = 7) were initially diagnosed possible ALS or Parkinsonian syndrome, respectively whereas none of SD or PNFA patients had a concomitant ALS or Parkinsonian syndrome.

### 3.2. Age of Onset and Family History

Age of onset ranged from 20 to the eighth decade. Patients with bvFTD tended to have a younger age of onset compared to patients with PNFA or SD, but this was not statistically significant as shown in Table 1 (*F* = 2.523, *P* = 0.096). Approximately 45.7% of bvFTD patients (*n* = 16), 37.5% of PNFA patients (*n* = 3), and 66.7% of SD patients (*n* = 4) were diagnosed before the age of 65. Overall, more than 47% FTD patients had the disease before 65. Seven bvFTD cases, but not from the other two groups, reported family histories.

### 3.3. Gender and Educations

Female patients comprised of 54.3%, 25%, and 50% of the bvFTD, PNFA, and SD subtypes, respectively. No significant differences in gender were demonstrated by crosstab chi-square analyses by sites and diagnostic subgroups (Fisher exact test, *P* = 0.387), although there appeared to be less females in the PNFA subgroup. Level of education varied greatly among patients with FTD, some were university graduates, and some were illiterate.

### 3.4. MMSE Score at First Visit

Only 19 of the 49 cases were presented with an MMSE score (9 with bvFTD; 6 with SD; 4 with PNFA group). A one-way analysis of variance showed no significant difference (13.9 ± 6.7; 15.3 ± 9.6; 15.7 ± 10.7) between diagnostic subgroups (*F* = 0.092, *P* = 0.91).

### 3.5. Neuropathological Diagnosis and Survival Curve Analysis

We found no reports of genetic screening. There are three neuropathological studies reporting a total of five FTD cases [14, 21, 46].  Table 2 summarizes the neuropathological diagnostic breakdown and demographic characteristics for the five cases.

**Figure 1:**
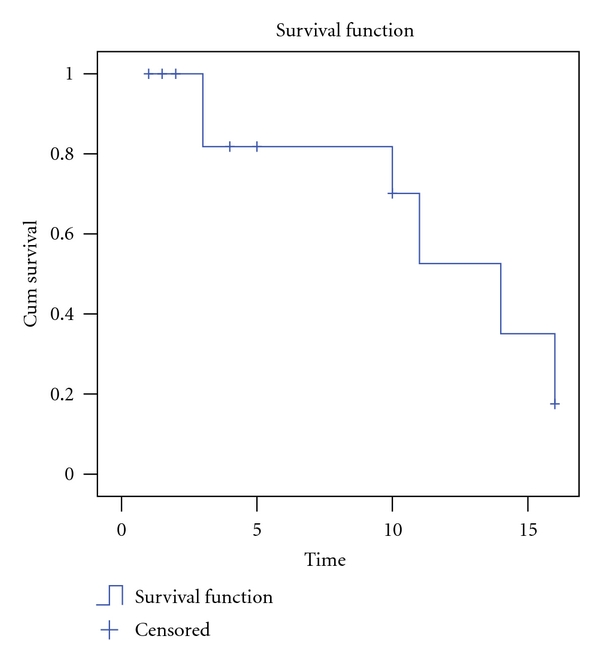
Survival curve of bvFTD in the present study.

 Median survival of 35 bvFTD cases presented in this study was 14 years from symptom onset (Figure 1). The survival curve demonstrated that most cases would progress for 10–15 years from time of diagnosis till death.

## 4. Discussion

The present study is the first, to our knowledge, to summarize demographic and clinical data from published cases in China according to the diagnostic criteria for FTD and its subgroups (bvFTD, PNFA, and SD). On the background of low diagnostic accuracy and lack of epidemiological data, this paper summarizes 49 cases from 24 articles and one small size retrospective study from multiple centers that demonstrated pathological features of FTD in China.

### 4.1. The Influences of Culture Background for Medical Service for FTD in China

The present study appeared to yield similar results compared to previous investigations from the west. BvFTD was the most common diagnostic subgroup and accounted for over half of all FTD diagnoses [6, 47]. SD and PNFA were less common and accounted for 12.2% and 16.3%, respectively. Interestingly, patients often favored neurology department or memory clinic over psychiatric clinic, although the first symptoms of FTD cases are frequently behavioral and psychiatric changes. This paradoxical phenomenon can be attributed to two potential factors. Firstly, families in the Chinese culture are often ashamed of psychiatric diseases, hence favoring seeking help in neurology clinics. Secondly, most specialists for cognitive disease are neurologists, and memory clinics or centers are generally run by neurology departments in China. Hence, the culture background may play an important role in the paradigm of delivering medical services to FTD patients in China, which is similar to Alzheimer's type dementia [48].

### 4.2. The Research History in Terms of FTD in China

The earliest clinical and neuropathological studies of dementia in China were by Tong and Wang in 1982 on Alzheimer's type dementia [49], and by Wang and her coworkers on Pick disease in 1993 [46]. The first confirmed case was a 93-year-old woman with ten-year history characterized by impairment of personal conduct and behavioral disorders with a positive family history. She died of acute respiratory failure, and autopsy results revealed typical Pick bodies. In retrospect, the earliest case might have been observed in 1960 but not recognised as such at the time [25]. Dr. Xu reported a 40-year-old male who presented with bizarre sexual behavior, as he bit off one third of his newly wed wife tongue on the first night of their wedding. He was referred to the psychiatric clinic, and a pneumoencephalography was performed with no abnormalities detected. He was suspected to have schizophrenia. Pneumoencephalography was repeated eight months later, revealing asymmetric frontotemporal lobe atrophy [25]. We suspect that FTD is not a rare disease in China, yet, to our surprise, there were only 49 FTD cases reported from China in the past 12 years. The overlap between FTD and Alzheimer's disease and psychiatric disorders, lack of neuropathological confirmation in most institutions in China, and the fact that clinicians are not fully aware of this disorder are the main causes for the underdiagnosis. Although brain MRI has been taken as a routine clinical examination for patients with dementia, most Chinese neurologists are short of neuropsychological training for FTD diagnosis.

### 4.3. The Current Features of FTD in China

Our study showed that features of FTD in China are largely similar to those reported in western countries with subtle differences [6, 38, 50]. As we gain more knowledge of FTD as a disease entity, we have observed that more FTD cases have been diagnosed compared to 20 years ago, and FTD has become one of most frequently diagnosed neurodegenerative dementias after AD in some memory clinics [51].

Similar to previous studies [6, 52], age of onset in FTD varied widely from the 20s to the 80s. Overall, 23 of the 49 cases had an age of onset at 65 years or younger accounting for 47% of the total. Patients with bvFTD appear to have a younger age of onset compared to patients with PFNA and SD. This is consistent with the average onset age of bvFTD, PFNA, and SD is 57, 65, and 59 from a joint British-Australian study [52].

Except in bvFTD (20% positive family history), family history does not appear to play an important role in PFNA and SD. Unfortunately, the education levels obtained for cases included in this study are ambiguous, as only the occupations are available but not the exact level of education received. Thus, we can only infer that the patients with FTD included a wide range, from professors to the illiterate.

The distribution of FTD across sexes appears roughly equal with some studies reporting a predominance in men although this has not been a consistent finding [6, 46, 53, 54]. Our study supported the equal sex distributions in bvFTD, but gender predominance in the other two subtypes could not be determined due to very small sample sizes.

Median survival in bvFTD in our study was 14 years from symptom onset which is consistent with other reports of 6–11 years [53–55]. ALS and parkinsonian syndrome can coexist with any of the FTD clinical variants but is most commonly associated with bvFTD [56, 57]. Our study found that 17% (*n* = 6) of the bvFTD patients also had possible ALS, whereas none of SD and none of PNFA patients had concomitant ALS. The bvFTD subgroup showed significant overlap with the atypical Parkinsonism associated with cortical basal degeneration (CBD).

Neuropathological diagnosis was carried out in 5 cases of bvFTD who showed gross atrophy of the frontal and anterior temporal lobes macroscopically. The neuropathological diagnoses included FTLD with tau-positive inclusions (FTLD-*τ*), FTLD with ubiquitin-positive and tau-negative inclusions (FTLD-U), and FTLD without tau-positive or ubiquitin-positive inclusions. The spectrums of neuropathological diagnosis are similar to previous studies [6].

### 4.4. The Progression of FTD Research in China

There is an increasing trend for FTD research in China in the past ten years. Unfortunately almost all research has focused on clinical aspects rather than neuropathology. Compared to Europe, North America, and Australia, studies of FTD in China are in a preliminary stage. Our review highlights the lack of neuropathological and genetic studies and of a unified clinical rating scale for FTD. For example, a myriad of single rating scales, including MMSE, Hasegawa Dementia Scale (HDS) [16, 31], Wechsler Adult Memory Scale (WAMS) [25], or the Chinese version of the revised Addenbrooke's Cognitive Examination (ACE-R) [58] were employed for screening in different memory clinics, which made comparison between cases difficult.

Therefore, a crucial development will be the application of consistent cognitive and behavioral rating scales for studies of FTD. This should be supplemented by genetic screening, particularly for mutations of the MAPT and progranulin in FTD patients with a strong family history. Longitudinal clinical followup would achieve better management of FTD and provide potential neuropathological diagnosis. We recommend the establishment of a multicenter work group in China which, we feel, would be a more efficient approach to epidemiological and clinical studies in the future.

In conclusion, this paper provides preliminary data to aid the understanding of the current situation of FTD in China. This review highlighted the importance of establishing a standardised protocol across different clinical/medical research sites to form a large combined cohort, in order to facilitate the exploring of epidemiological, clinical, and biological features of FTD, hence ultimately improving clinical diagnosis accuracy and quality of life for patients with FTD in the world including China.

## Figures and Tables

**Table 1:** Demographic variables by diagnosis.

Variable*∖*subgroups	bvFTD	PFNA	SD	Overall
MMSE score at the initial assessment	13.9 ± 6.7	15.3 ± 9.6	15.8 ± 10.7	14.7 ± 8.0
Age at onset (years)	53.8 ± 14.8	64.1 ± 10.1	63.8 ± 9.9	57.8 ± 13.8
Male/sex	16/35	6/8	3/6	25/49

**Table 2:** Characteristics by neuropathological diagnosis.

Neuropathological diagnosis	Age at onset (years)	Age at death (years)	Initial MMSE score	Family history
(1) FTLD-*τ*	60, 72	76, 86	N/A, 8	No
(2) FTLD-U	58	61	22–30	No
(3) FTLD without intracellular inclusions	67, 43*	78, 46*	N/A	No

N/A = not available; *Ubiquitin immunohistology examination was not performed on this case (with possibility of FTLD-U).
